# Predictors of therapy switching to high-efficacy disease-modifying therapies in patients with multiple sclerosis: a single center, retrospective, observational study

**DOI:** 10.3389/fneur.2025.1635618

**Published:** 2025-10-09

**Authors:** Gytis Makarevičius, Rasa Kizlaitienė, Gintaras Kaubrys, Nataša Giedraitienė

**Affiliations:** Faculty of Medicine, Institute of Clinical Medicine, Vilnius University, Vilnius, Lithuania

**Keywords:** multiple sclerosis, prognosis, symptoms, therapeutic choice, high-efficacy therapy

## Abstract

**Object:**

The treatment of multiple sclerosis (MS) with high-efficacy disease-modifying therapies (HE-DMTs) may lead to better long-term outcomes for patients. There is an ongoing debate about which patients should initially start with these treatments. The objective of this study was to assess the first symptoms at the time of MS diagnosis and to identify independent predictors of treatment switching to HE-DMTs in MS patients within 5 years after diagnosis.

**Materials and methods:**

A single-center retrospective, observational study was conducted at tertiary MS center Vilnius University Hospital Santaros Klinikos, Lithuania. 319 patients newly diagnosed with relapsing MS who were initially treated with MS platform therapy between 2010 and 2019 were included.

**Results:**

During the disease course, 26.65% of patients were switched from platform therapy to HE-DMTs within 5 years of follow-up. Factors associated with the need to switch therapies were younger age (*p* < 0.001), shorter disease duration (*p* < 0.001) and higher progression index (*p* < 0.001) at diagnosis, lower initial EDSS (*p* = 0.003) and the presence of cerebellum and/or brainstem symptoms (*p* = 0.047). Younger age, shorter disease duration and cerebellar/brainstem presentation at diagnosis remained statistically significant after logistic regression analysis.

**Conclusion:**

Younger age, shorter disease duration and cerebellar/brainstem presentation at diagnosis were consistently associated with the need to escalate platform.

## 1 Introduction

Multiple sclerosis (MS) is a chronic, progressive, immune-mediated and neurodegenerative disease which causes progressive neurological damage and is the most frequent cause of non-traumatic disability in young adults (1–3). A large portion of MS patients are young, working-age adults, which is one of the leading reasons for the significant economic burden (1). Although there is still no curative treatment, neurological disability progression could be slowed with a growing number of disease-modifying therapies (3). Based on efficacy, these treatments are generally categorized into two major groups: low/moderate-efficacy disease-modifying therapies (LE-DMTs) and high-efficacy disease-modifying therapies (HE-DMTs), with ongoing debate on who should receive which treatments and about the optimal timing of initiation (2, 3). Early MS treatment with HE-DMTs significantly reduces the risk of disability progression compared to delayed treatment or escalation from LE-DMTs (2, 4–7). Studies show that early use of HE-DMTs is linked to better long-term outcomes, including reduced relapse rates and slower disease progression (6, 8, 9). The use of HE-DMTs, especially anti-CD20 drugs, also reduces the need to switch between different medications (10). The first therapeutic choice is crucial for the prognosis of MS patients. Suboptimal treatment is associated with an increased risk for relapses, developing new brain lesions, higher risk of disease progression and achieving the Expanded Disability Status Scale (EDSS) score 3.0 and 6.0 earlier (11). Thus, the MS treatment paradigm has recently been shifting toward earlier initiation of HE-DMTs (12–14). However, MS is a heterogeneous disease with considerable differences in disease course for individual patients (15). In addition, early high-efficacy therapies carry higher risks of side effects and require more intensive – and usually more burdensome – monitoring, which may not be justified for patients with mild disease courses (2, 8, 16). A strategy of universal HE-DMT initiation, followed by subsequent treatment de-escalation, may not be optimal, as there still remains a need to identify high-risk patients for whom de-escalation may not be appropriate (17). Current disease progression surveillance and escalation strategies may not be adequate, as up to 12 percent of patients will subsequently require HE-DMTs within 5 years of treatment (18). Thus, an early prediction of disease course (high vs. low risk) and appropriate, individualized decision on the first treatment, considering disease severity, risk factors, and patient preferences, is crucial (19, 20). In recent years, attempts have been made to identify risk factors associated with treatment switching (19, 21). A dynamic scoring system to improve treatment switching decisions was even proposed by a French study (22). Nevertheless, considering recent findings, the focus should shift from gradually switching treatment based on disease progression to identifying high-risk patients at the diagnosis to initiate HE-DMTs earlier. A need for evidence emerges to identify the crucial factors determining which people with MS should initially start with HE-DMTs.

The objective of this retrospective, observational study was to assess the first symptoms at the time of MS diagnosis and to identify independent predictors of treatment switching to HE-DMTs in MS patients within 5 years after diagnosis, suggesting that an initial HE-DMTs approach might have been more appropriate.

## 2 Materials and methods

### 2.1 Study design and data collection

A single-center, retrospective, non-interventional case series study using real-world data was conducted at tertiary Multiple Sclerosis (MS) Center Vilnius University Hospital Santaros Klinikos (VUHSK), Lithuania. Data were obtained from the VUHSK MS Center registry, which contains information on patients diagnosed with MS at the VUHSK MS Center from the year 2010 to the present. The Vilnius MS Center registry currently includes approximately 1,500 patients diagnosed with MS, of whom about 1,000 are regularly followed at the Centre. Nearly half of the registered patients were enrolled within the past 5 years (2020 to 2025). Patients typically attend clinical visits every 1–3 months. The registry collects individual-level data on demographics; results from specific diagnostic procedures (such as cerebrospinal fluid analysis and evoked potentials); clinical evaluations conducted at each visit (based on the EDSS); as well as information on treatment and relapses (including their dates and whether corticosteroid treatment was administered). We have retrieved information from the registry regarding initial patient data (at the time of diagnosis) and the 5 years following the diagnosis. Patients newly diagnosed with relapsing MS (according to McDonald criteria) at VUHSK MS Center between 2010 and 2019 and who were initiated platform therapy at the diagnosis were included in the analysis. Only those subjects with complete datasets (age, sex, EDSS scores, reported symptoms and treatments) were included. Patients who stopped treatment within 5 years of follow-up were also excluded. Those patients who were started on HE-DMTs immediately after the MS diagnosis and those patients whose EDSS score at diagnosis exceeded six (thus, ineligible for HE-DMTs on the regulations of the Ministry of Health of Lithuania) were excluded. The primary outcome was initiation of HE-DMTs within 5 years after the diagnosis (±3 months). Baseline EDSS score was defined as that recorded at the diagnosis. Final EDSS score was taken 5 years later for every patient (±3 months). EDSS scores were confirmed at the subsequent visit to avoid misclassification due to temporary fluctuations. Confirmed disability progression was defined as an increase in the EDSS score of at least one point. Age was recorded at the time of diagnosis.

In Lithuania, approvals for MS treatments are regulated following the regulations of the Ministry of Health of Lithuania. Patients are generally started on platform therapy. Only those who experience two or more disabling exacerbations within 1 year and who either present with at least one gadolinium-enhancing lesion on brain magnetic resonance imaging (MRI) or demonstrate an increased number of T2 lesions compared with the previous MRI are eligible to receive HE-DMTs as first-line therapy. Patients initially started on platform therapy may later be escalated to HE-DMTs if they experience at least one relapse per year with new or active lesions on brain MRI, or two relapses per year while on first-line therapy. The prescription of high-efficacy therapies is not restricted by patients’ age or disease duration. The only regulatory limitation is disability status: treatment may be initiated only if the EDSS score does not exceed 6. In our study, treatment escalations occurred in accordance with these national regulations.

### 2.2 Definitions and variables

MS treatment with interferons, glatiramer acetate, dimethyl fumarate and teriflunomide was defined as platform therapy. High efficacy therapies for MS were fingolimod, cladribine, natalizumab, alemtuzumab and ocrelizumab. These high efficacy therapies are based on the regulations of the Ministry of Health of Lithuania. The Multiple Sclerosis Severity Score (MSSS) and the Age-Related Multiple Sclerosis Severity Score (ARMSSS) were calculated as previously defined (16, 23). We used ms.sev package for R to compose new variables (MSSS and ARMSSS) from initial EDSS score, age and disease duration. Updated global MSSS and global ARMSSS references were chosen. The progression index (PI) was calculated by dividing the EDSS score at diagnosis by the duration of the disease at diagnosis in years. Disease duration was defined as the period of time (in years) from the onset of any self-reported MS-related symptoms to the time of diagnosis. First clinical symptoms were grouped according to predefined neurological functional systems (e.g., cerebellar, brainstem, and pyramidal) following EDSS guidelines.

### 2.3 Statistical analysis

Descriptive statistics were employed to summarize demographic and multiple sclerosis related information. Dichotomous and nominal data were presented as raw numbers and percentages of the total. Numerical data was summarized using mean ± standard deviation, median and interquartile range as appropriate depending on distribution. Statistical analysis was performed in R software (version 4.1.2). A significance level of <0.05 was chosen. The Lilliefors test was used to test for normality, the two-variances *F*-test for homogeneity of data. The Student’s *t*-test was utilized to compare means of normally distributed data and Student’s *t*-test non-parametric alternative (Mann–Whitney *U* test) was utilized to compare differences in ordinal and interval data. For dichotomous variables, chi-square analysis was employed. As the data contained collinear variables (e.g., disease duration at the time of diagnosis/PI and EDSS/MSSS/ARMSS) we applied elastic net regularized regression method with an alpha of 1.0 to identify predictors associated with therapy switching. Selected variables were included into final binary logistic regression model. The model was checked for multicollinearity using Variance Inflation Factor (VIF) and the Area under the curve (AUC) of the Receiver Operating Characteristic (ROC) curve and Pseudo R^2^ (McFadden) were chosen for model performance.

## 3 Results

**Figure 1:**
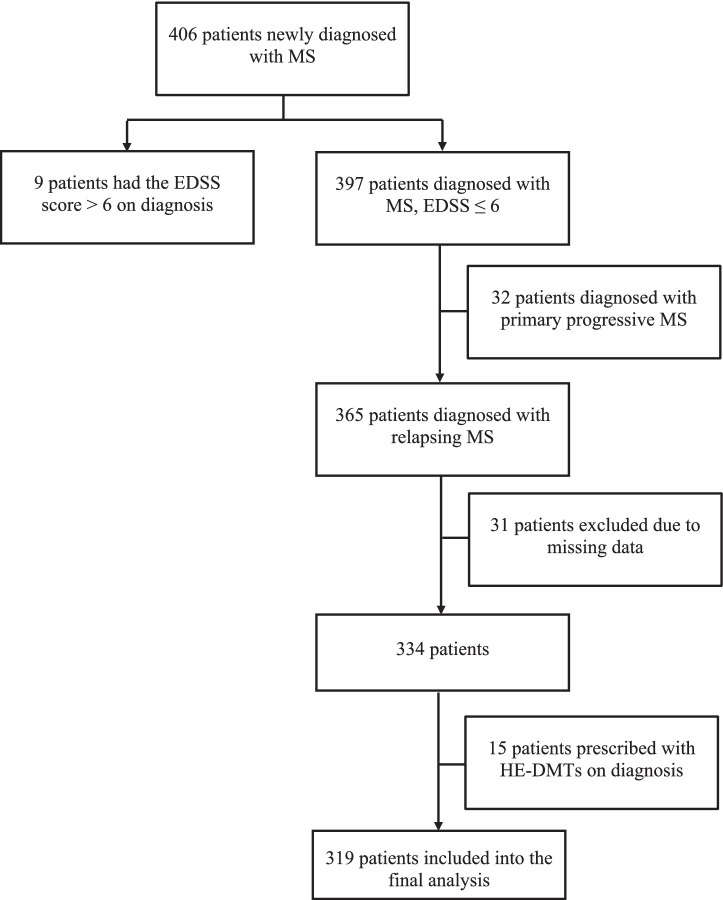
Flowchart of sample inclusion criteria. MS, Multiple sclerosis; EDSS, Expanded Disability Status Scale; HE-DMTs, high-efficacy disease-modifying therapies.

**Figure 2:**
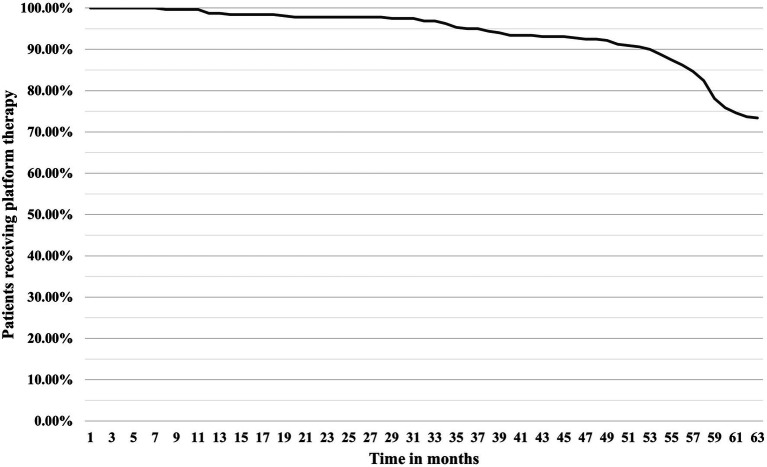
Time-to-switch curve from platform therapy to HE-DMTs. HE-DMTs, high-efficacy disease-modifying therapies.

A total of 334 patients were identified in our study according to the inclusion criteria (Figure 1). Of these, 15 (4.49%) were initially started on HE-DMTs and were therefore excluded, leaving 319 patients for the final analysis (Figure 1). The majority were women (221, 69.28%) with a mean age of 45.48 (± 10.08) years. The median duration of disease (first self-reported MS symptoms) at the time of diagnosis was 5.96 years (1.92, 11.02) with most prevalent brainstem (147, 46.08%) and supratentorial (113, 35.42%) symptoms at the time of diagnosis. Optic symptoms and spinal cord symptoms were reported less frequently (80, 25.08% and 52, 16.30%, respectively). The initial median EDSS score was 2.5 (2.0, 3.0) which progressed within 5 years to a median of 3.0 (2.0, 4.0). During the disease course, 85 of 319 patients (26.65%) were switched from platform therapy to HE-DMTs with median time to escalation of 56 months (43, 59) (Figure 2). The factors associated with the need to switch from platform to HE-DMTs were younger age (41.15 vs. 47.05 years, *p* < 0.001), shorter disease duration (2.41 vs. 7.77 years, *p* < 0.001) and higher progression index (0.92 vs. 0.35, *p* < 0.001) at diagnosis, lower initial EDSS (2.0 vs. 2.5, *p* < 0.001) and the presence of cerebellum and/or brainstem symptoms (55.30% vs. 42.70%, *p* = 0.047). Sex, the scores of MSSS and ARMSS were not statistically significantly associated with treatment switching (Table 1).

**Table 1:** Predictors of treatment switching to HE-DMTs in MS patients.

Predictors	Patient groups		
	Non-switchers (*n* = 234)	Switchers (*n* = 85)	*p*-value
Age – mean (±SD)	47.05 (±10.06)	41.15 (±8.83)	**<0.001**
Sex – *n* (%)			0.604
Female	164 (70.10%)	57 (67.10%)	
Male	70 (29.90%)	28 (32.90%)	
Baseline EDSS – median (Q1, Q3)	2.5 (2.0, 3.5)	2.0 (1.5, 2.5)	**<0.001**
EDSS after 5 years – median (Q1, Q3)	3.0 (2.0, 4.0)	3 (2.5, 4.0)	0.501
EDSS net increase in 5 years – median (Q1, Q3)	0.0 (0.0, 0.5)	1.0 (0.0, 1.5)	**<0.001**
Baseline ARMSSS – median (Q1, Q3)	3.89 (2.41, 5.21)	4.01 (2.51, 5.21)	0.696
Baseline MSSS – median (Q1, Q3)	4.51 (3.13, 5.95)	4.51 (3.90, 6.35)	0.257
Disease duration in years – median (Q1, Q3)	7.77 (2.65, 12.14)	2.41 (0.51, 5.88)	**<0.001**
Symptoms at diagnosis – *n* (%)			
Spinal cord	36 (15.4)	16 (18.8)	0.462
Cerebellum and/or brainstem	100 (42.70)	47 (55.30)	**0.047**
Supratentorial	89 (38.0)	24 (28.2)	0.106
Optic	61 (26.10)	19 (22.4)	0.499
PI – median (Q1, Q3)	0.35 (0.21, 0.79)	0.92 (0.36, 3.49)	**<0.001**

HE-DMTs, high-efficacy disease-modifying therapies; EDSS, Expanded Disability Status Scale; ARMSSS, Age Related Multiple Sclerosis Severity Score; MSSS, Multiple Sclerosis Severity Score; PI, Progression index (Baseline EDSS/Disease duration in years). Bold values indicate statistical significance (*p* < 0.05).

Despite high intensity treatment, the disease course of patients who required switching therapy was unfavorable. The disease progressed with a median of 1 EDSS score within 5 years compared to 0 of non-switcher group (*p* < 0.001).

We analyzed the patient characteristics (age, sex, disease duration, progression index, EDSS, MSSS, ARMSSS scores, first neurological symptoms at the time of diagnosis) with elastic net regression model as this model helps both with variable selection and stabilization of model with many interdependent variables. The dependent binary variable in the model was therapy switching (continuing platform therapy or switching to HE-DMT within 5 years). The elastic net regression model retained 5 variables as significant predictors: age, disease duration, EDSS at diagnosis, cerebellum and/or brainstem symptoms and supratentorial symptoms. The final binary model (Table 2) achieved AUC of 0.739, indicating acceptable discrimination. McFadden’s Pseudo R^2^ was 0.123 indicating weak-moderate fit. In the model, age, disease duration at diagnosis and the presence of cerebellum and/or brainstem symptoms at diagnosis maintained statistical significance. Every increase in age and disease duration at diagnosis by a year was associated with approximately 4% and 8% decreases, in the need to switch therapy to HE-DMT, respectively, the presence of initial cerebellum and/or brainstem involvement increased the odds by 77%.

**Table 2:** Final binary logistic regression model of factors associated with platform therapy switching to HE-DMTs.

Predictor	β (SE)	OR (95% CI)	*p*-value
Intercept	1.900 (0.71)	6.70 (1.67, 26.90)	**0.007**
Age	−0.044 (0.02)	0.96 (0.93, 0.99)	**0.005**
EDSS	−0.270 (0.15)	0.76 (0.57, 1.03)	0.073
Disease duration	−0.080 (0.03)	0.92 (0.87, 0.98)	**0.005**
Cerebellum and/or brainstem symptoms	0.573 (0.28)	1.77 (1.03, 3.07)	**0.040**
Supratentorial symptoms	−0.269 (0.30)	0.76 (0.43, 1.37)	0.369

HE-DMTs, high-efficacy disease-modifying therapies; EDSS, Expanded Disability Status Scale. Bold values indicate statistical significance (*p* < 0.05).

Additionally, we found significant association between spinal cord symptoms and shorter disease duration at the diagnosis (6.44 vs. 4.01 years, *p* = 0.004), higher progression index (0.37 vs. 0.71, *p* < 0.001) and higher MSSS score (4.43 vs. 5.52, *p* = 0.003). Although older age (45 years or older) was associated with a higher EDSS after 5 years of observation (3.5 vs. 2.5, *p* = 0.004) and a longer disease duration (9.06 vs. 3.52 years, *p* < 0.001), it was not significantly associated with disease progression (EDSS increase by more than 0.5) over a 5-year period (27.3% in 45 years or older patients vs. 35.7% in younger than 45 years old, *p* = 0.104) and was noted for overall lower progression index (0.29 vs. 0.60, *p* < 0.001). There were no statistically significant symptoms association with age, except for optic symptoms which were more common in younger patients (30.5% vs. 20.0%, *p* = 0.030).

## 4 Discussion

Here, we report our single-center, retrospective, non-interventional case series study using real-world data from 319 patients with relapsing MS. In our cohort, initiation of HE-DMTs as first-line therapy was infrequent (4.49% vs. 17.60%), and escalation to such therapies occurred after longer median intervals (56 months vs. 29 months) compared with a study conducted in the United Kingdom with a comparable follow-up duration – a pattern that may be attributable to more stringent national prescribing regulations and prescription trends evident in some other European countries as well (13, 18, 24). However, the percentage of patients receiving HE-DMTs within 5 years was similar in both cohorts (29.94% vs. 27.36%) (18). We also found that in our studied population younger age, shorter overall disease duration and cerebellum and/or brainstem symptoms at diagnosis were consistently associated with switching from platform therapy to HE-DMTs within 5 years of disease diagnosis. Although lower EDSS at diagnosis and higher progression index were statistically significantly associated with the need to switch therapies in primary analysis, they did not maintain consistent statistical significance after logistic regression analysis. In addition, the effect size determined for age and disease duration was quite low (4% and 8% every year, respectively). This reflects a previously described issue advocating for broader use of HE-DMTs (25).

In previous studies age, treatment failure, intolerance and side effects, the degree of MS disability were the predictors most consistently reported to be associated with treatment switching (10, 19, 26–32). More frequent MRI use, which detects subclinical MS activity more sensitively than clinical assessment alone, has also been associated with treatment switching – particularly among patients on LE-DMTs – but not among those receiving HE-DMTs (33). However, the findings in this area are not entirely consistent. For example, a noteworthy analysis of a huge cohort has determined female sex, older age, higher EDSS, longer disease duration at treatment initiation to be independent factors of treatment switching (19). On the other hand, age of <26 years in one study was associated with failure of first line treatment (29), younger age in general was associated with switching therapies in some other studies (10, 30, 31) and older age was associated with less frequent occurrence of at least one clinical relapse (11). Younger patients with shorter disease duration and lower EDSS at treatment switching seem to benefit more (22). In addition, de-escalation from HE-DMTs to platform therapy in younger patients has been associated with an increased risk of inflammatory disease activity following treatment modification (34). By contrast, in older patients the risk appears to be lower (35). These discrepancies between studies could be caused by different methodologies (evaluating individual medication switching vs. medication group switching vs. general disease course, etc.). Pregnancy, intolerance and side effects of medication could also affect treatment switching, especially considering switching made within the same efficacy group of DMTs. Thus, treatment switching predictors determined by some studies cannot always be translated to predict which patients are at higher risk and could benefit from early HE-DMTs. The key difference in our study was that we evaluated patients who were prescribed platform therapy to see who will be switched to HE-DMTs. In Lithuania, MS treatment is regulated by the Ministry of Health and switching to HE-DMTs is permitted only when predefined criteria are met. This way, the sole reason for switching in our cohort was active disease progression and ineffectiveness of platform therapy (patients are switched to HE-DMTs if they experience at least two relapses per year or one relapse per year with new or active lesions on MRI) within 5 years of treatment. This is also emphasized by EDSS progression in the switchers group, which was statistically significantly higher than in non-switcher group. Moreover, our findings that younger age and shorter disease duration are associated with treatment switching could be explained by the pathogenesis of MS. A two-stage model of disability progression was previously proposed with the initial phase being active immune mediated inflammation that is followed by the second phase in which disability progression is independent of inflammatory markers (36). It was also shown that there is a decrease in T cell activation markers with disease duration which could indicate transitioning from a predominantly inflammatory profile to a more chronic, degenerative state (37). Similarly, MS-related inflammation is more evident in younger patients decreasing as patients age (2). This phenomenon could be explained by the concept of immunosenescence, which describes the gradual deterioration of the immune system associated with aging, a process which is accelerated in patients with autoimmune disease (38). As individuals age, their immune systems undergo significant changes, leading to reduced adaptive immune function, which is believed to drive MS relapses (38). However, later stages of disease are believed to be mediated by compartmentalized innate immune responses, a process linked to immunosenescence and older age (38, 39). There is also some evidence that immunomodulatory DMTs are more effective in early stages of the disease and for younger patients, as well as immunomodulatory treatment of secondary progressive multiple sclerosis (which could be simply viewed as later stage of MS disease process – combining both more advanced age and longer disease duration) is only efficient in patients with active disease (40, 41). Further solidifying the claim that autoinflammation is most potent in younger patients with shorter disease duration. Thus, younger age and shorter disease duration remain the key risk factors of treatment switching to immunomodulatory HE-DMTs.

The other interesting aspect to investigate is the clinical symptoms of MS at diagnosis and subsequent risk for treatment switching. Spinal, cerebellar and/or brainstem presentation were previously described of being factors associated with poorer disease outcomes (42, 43). Spinal cord lesions were also reported to be associated with increase switch rates (30). Interestingly, the presence of cerebellum and/or brainstem symptoms was also found to be associated with treatment switching in our study. However, spinal presentation was not shown to be statistically significantly associated with treatment switching despite exhibiting an increased disease progression index at diagnosis. This could be explained by the lack of sufficient statistical power when distributing patients to different symptom groups. There is a need for more research to ascertain the relationship between initial clinical symptoms and risk of treatment switching to HE-DMTs in MS patients.

As previously mentioned, the degree of MS disability (most frequently expressed as the score of EDSS) was also linked to treatment switching. As in age studies, there are also some inconsistencies. Some studies identify higher EDSS at treatment start as a risk factor for treatment switching (19, 29, 30), while others do not (32). In contrast, our study found the opposite trend – there were lower frequency of switching to HE-DMTs in patients with higher initial EDSS. However, after performing logistic regression analysis, EDSS was not statistically significantly associated with treatment switching. Other MS severity scores (MSSS, ARMSSS) failed to show any significant associations even in primary analysis despite being argued to better describe MS related disability (44). As both scores use reference population data, they might not be always applicable to certain populations (in this case Lithuanian). Nevertheless, MS disability is generally lower in younger patients and in the initial stage of the disease, progressing over time, thus, MS severity scores might not be the best tools to guide decision making regarding initiation of HE-DMTs.

To our knowledge, this is the first study evaluating which factors are specifically associated with treatment switching from platform MS therapy to HE-DMTs, as previous studies mainly focused on switching between individual medication. However, this study has several limitations. First, we could not provide initial MRI data to include in the predictor analysis. Additionally, the retrospective nature of the study limited our ability to adjust for previously described risk factors associated with MS exacerbations and progression, such as vitamin D deficiency and supplementation, Epstein–Barr virus infection, obesity, and smoking (45). Moreover, patients with EDSS >6 were excluded, and therefore outcomes in patients with more aggressive MS phenotypes were not assessed. The study also lacked sufficient statistical power to detect statistically significant changes by some different symptom groups. Some subjectivity may remain in symptom classification, which is also a limitation of this study. Lastly, although in Lithuania the escalation of HE-DMTs is strictly regulated by the Ministry of Health and occurred only in the described settings, in theory there could have been cases in which patients met the criteria for escalation but it did not occur for some reason (e.g., the patient refused escalation). We were unable to report these cases, which adds to the limitations of our study.

## 5 Conclusion

Younger age, shorter disease duration and cerebellar/brainstem presentation at diagnosis were consistently associated with the need to escalate platform MS treatment to HE-DMTs, although the effect sizes for age and disease duration were small. Given recent evidence favoring early HE-DMT initiation over escalation, and the limited ability to predict long-term outcomes at onset, earlier use of HE-DMTs may be warranted, particularly in younger patients, those with shorter disease duration, and those presenting with cerebellar or brainstem symptoms. Patients maintained on platform therapy should be closely monitored clinically and with more frequent MRI assessments. More precise prognostic models, potentially incorporating current and yet-to-be-developed biomarkers, are needed to better guide precision medicine.

## Data Availability

The raw data supporting the conclusions of this article will be made available by the authors, without undue reservation.
